# The Fluoride Ion Release from Ion-Releasing Dental Materials after Surface Loading by Topical Treatment with Sodium Fluoride Gel

**DOI:** 10.3390/jfb14020102

**Published:** 2023-02-13

**Authors:** Marija Kelić, Domagoj Kilić, Katarina Kelić, Ivana Šutej, Matej Par, Kristina Peroš, Zrinka Tarle

**Affiliations:** 1 School of Dental Medicine, University of Zagreb, Gundulićeva ul. 5, 10000 Zagreb, Croatia; 2 Dental Clinic Zagreb, Perkovčeva ul. 3, 10000 Zagreb, Croatia

**Keywords:** recharge potential, topical treatment, ion-releasing dental materials, adhesive systems

## Abstract

The study aimed to investigate the rechargeability of ion-releasing dental material specimens immersed in distilled water for 25 months, which depleted their ion-releasing ability. Four restorative dental materials (alkasite composite, giomer, glass-ionomer, and composite material) presented with 24 specimens were studied after topical treatment with a concentrated fluoride gel. The effect of resin coating on the ion uptake and release was investigated on additional 42 specimens of restorative dental materials with coatings. The composite materials were coated with two adhesive systems, whereas the glass-ionomer was coated with the special coating resin. After topical fluoride exposure, ion release and specimen mass were measured at 1, 2, 3, 4, 5, 6, 7, and 14-day intervals using an ion-selective electrode and an analytical balance, respectively. The cumulative fluoride levels for the uncoated specimens of alkasite composite were significantly higher than those of giomer and glass-ionomer cement, with no statistically significant difference between the latter two materials. The conventional composite had the lowest cumulative concentration of fluoride ions (*p* < 0.05). The adhesive systems affected the fluoride recharge and reduced the ion concentrations absorbed by the specimens. Specimens coated with universal adhesive showed significantly higher ion release compared to universal fluoride-releasing adhesive or special coating resin for glass-ionomers (*p* < 0.05). No statistically significant change in specimen mass was observed during the 14-day period. Surface coating with adhesive systems as well as special coating resin for glass-ionomers affects the fluoride recharge process.

## 1. Introduction

The topical application of fluoride is considered an important factor in preventing the development of caries and interrupting and stopping the further demineralization of teeth [1]. The stringent requirements placed on restorative materials include the ability to release fluoride ions and to subsequently recharge with fluoride ions. The restorative material can store fluoride ions when exposed to a medium enriched with fluoride ions after previously releasing a certain number of ions from its structure [2]. The material structure, the product type, and the concentration of fluoride ions to which the material is exposed are factors that determine the number of fluoride ions that the material can receive [3,4,5,6]. Ion-releasing restorative materials have a relatively well-explored fluoride release time [7]; hence, the question of interest is whether the recharge process may be clinically relevant as the material genuine fluoride release. 

The permeability, composition, porosity, and surface retention of ions in dental restorative materials affect the recharge capacity [3,8,9,10]. More permeable materials can store fluoride ions deeper in their structure, whereas impermeable materials adsorb ions mainly on their surface [11]. Highly concentrated fluoride solutions, gels, and varnishes can contain up to 44,800 ppm F^−^ [12]. The cariostatic concentration of fluoride ions released from the restorative material varies with time. The initial release of a large number of fluoride ions, known as the “burst effect,” is observed within 24 h of mixing the glass-ionomer cement. The initial burst is followed by a slower continuation of the release until the level of released ions stabilizes [13,14]. Some in vitro studies have shown that the long-term release of fluoride ions from glass-ionomer cement persists for several months to three years [15,16,17]. Although, there is no published data on rechargeability of materials aged more than 18 months.

The proposed mechanism of initial ion release from the glass-ionomer begins with the contact of the glass filler particles with the polyacrylic acid during the setting reaction of the cement. The process is initially rapid and involves the release of surface ions into the medium surrounding the material. The slower process of diffusion of fluoride ions through pores and fracture cracks indicates a continuous release of ions from the material [17]. Dental materials interact with the ions by which they are surrounded: physical adsorption of ions, chemical bonding with the surface of the material, or, more commonly, ions are retained at the surface of the material due to the simultaneous action of both bonding methods [18]. Additionally, in study by Williams et al. [15] artificial saliva or deionized water were used and compared, with slightly reduced ion release affected by artificial saliva.

The limited number of in vitro studies claim that all materials can be recharged with fluoride ions to some extent, showing that glass-ionomer and resin-modified glass-ionomer cements have a higher value in terms of fluoride ion re-release compared to composite materials and that the tested materials show the ability to recharge after topical treatment with sodium fluoride solution, as concluded by Rai et al. [19] as well. Although, there is no published data on recharge treatment protocol with topical fluorides in very high concentration, it was found that the ability of the materials to absorb more ions during the “recharge” treatment was proportionally related to the number of ions released during the initial release phase. This means that materials that release more ions are also able to absorb more ions during the recharging treatment [2,16,17,20,21].

However, there are findings of some materials without prior ion release capability that were able to release more ions after the recharge protocol compared to some glass-ionomer materials [3]. Although previous studies indicated the superiority of glass-ionomer cements in terms of rechargeability compared to giomer materials, according to Rai et al. [19], newer materials such as the “alkasite” composite show the ability to release more fluoride ions than glass-ionomer cement.

The recharge process and re-release of fluoride ions seems to be thoroughly investigated. However, there remains a lack of research on material specimens aged more than 18 months, treatment protocol with higher concentration of topical fluoride, and the effect of adhesive systems on recharge and re-release.

This study aimed to expose glass-ionomer, giomer, and composite restorative materials to a highly concentrated fluoride-containing gel and investigate their rechargeability of each after ageing of 25 months. The secondary aim was to test the influence of specimen surface coating with adhesive systems or a glass-ionomer coating resin on the recharge process. Our first null hypothesis is that there is no difference among the materials in the uptake and the amount of re-released fluoride ions as well as between coated and uncoated specimens The second null hypothesis is that there is no statistically significant change in specimen mass during the 14-day period. 

## 2. Materials and Methods

Four restorative dental materials were investigated: an “alkasite” composite material (Cention, Ivoclar Vivadent, Schaan, Lichtenstein), a giomer material (Beautifil II, Shofu Dental, GmbH, Ratingen, Germany), a conventional glass-ionomer cement (GIC) (GC Fuji IX Extra, GC, Tokyo, Japan) that served as a positive control and a conventional composite (Filtek Z250, 3M Deutschland GmbH, Neuss, Germany) that served as a negative control. Materials were selected to represent different material groups on the basis of availability on the market. The adhesive systems used to coat resin composite specimens included a universal one-bottle adhesive system (G-aenial™ Bond, GC Europe, Leuven, Belgium) as an adhesive without fluoride, and a universal fluoride-releasing adhesive system (Clearfil Universal Bond Quick, Kuraray Europe, Hattersheim am Main, Germany) as a fluoride positive adhesive. The glass-ionomer specimens were coated using Fuji IX Coat GP (GC Europe, Leuven, Belgium). The list of investigated materials with their composition provided by the manufacturers is presented in Table 1.

**Figure 1:**
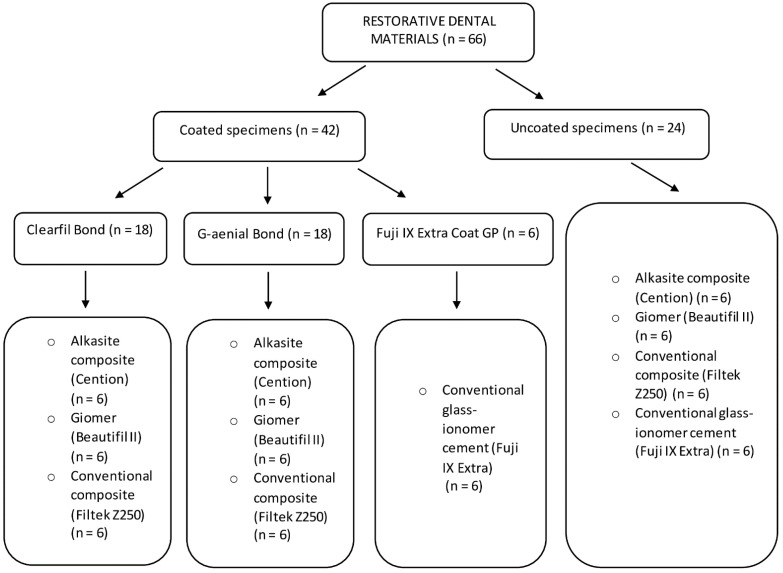
Study design and division of the tested materials into experimental groups. n = number; Clearfil Bond—an universal fluoride—releasing adhesive system; G-aenial Bond—an universal one—bottle adhesive system; Fuji IX Extra Coat GP—glass-ionomer coating.

Resin composite specimens were prepared in two groups. One group consisted of uncoated specimens, whereas the specimens in the other group were coated with adhesive systems. The latter was divided into two subgroups coated with either a universal adhesive (G-aenial™ Bond, GC Europe, Leuven, Belgium) or a fluoride-releasing adhesive (Clearfil Universal Bond Quick, Kuraray Europe, Hattersheim am Main, Germany). Specimens of glass-ionomer cements were divided into two groups: the uncoated group and the group coated with a glass-ionomer coat (GC Fuji Coat LC, GC Europe, Leuven, Belgium). The study design and the allocation of specimens into experimental groups is shown schematically in Figure 1. Each group comprised 6 specimens (n = 6).

The specimens used in this study had previously been stored in distilled water for 25 months. These specimens were examined in a previous study [22] in which their preparation, treatment, and measurements were described in detail. Specimens were polymerized using an LED curing unit (Bluephase G2, Ivoclar Vivadent, Schaan, Liechtenstein) with a nominal intensity of 1200 mW/cm^2^ for 20 s on each side. Samples were immersed separately in polystyrene individual vials containing 5 mL of deionized water at 37 °C and evaluated for fluoride content release at time intervals within 168 days until the fluoride release reached undetectable values. After this part of the trial was completed, each specimen was individually placed in 5 mL of distilled water in sealed polystyrene cup and stored in a dry, closed, dark chamber.

Each specimen was assigned a flag with its designation, and the mass of the specimens was measured with a laboratory scale (Analytical balance ABS-N/ ABJ-NM, KERN, Balingen, Germany). Five milliliters of deionized water (T.T.T., Sveta Nedelja, Croatia) was pipetted into the polystyrene cups, and the specimens were placed into the cups, sealed, and placed in an incubator (Domel, Železniki, Slovenia) at 37 °C. After 24 h, the specimens were removed from the incubator, dried with paper towels (TM-Horeca, Ljubljana, Slovenia), and their masses were measured again. The specimens were treated with a concentrated fluoride gel (NaF of 12,300 ppm F^−^, pH 5.5, Miradent Mirafluorr-gel; Hager & Werken GmbH & Co. KG, Duisburg, Germany) for 4 min with agitation and then rinsed with distilled water (T.T.T., Sveta Nedelja, Croatia) for 20 s. This was the one and only fluoride treatment point. The specimens were immersed in 5 mL of deionized water and placed back in the incubator at 37 °C. The release of fluoride ions was measured at intervals of 1, 2, 3, 4, 5, 6, 7, and 14 days. At each indicated interval, the specimens were treated in the same manner. Specimens were removed from the incubator, dried with paper towels, and their mass was measured. Then, the specimens were returned to freshly pipetted 5 mL deionized water, stored in sealed cups, and placed back in the incubator at 37 °C until the next time interval.

At each time interval, 4.5 mL of the immersion solution was separated and 0.5 mL of buffer solution (TISAB III; Thermo Fisher Scientific, 22 Alpha Road, Chelmsford, MA 01824, USA) was added. The amount of fluoride ions released from dental restorative materials after topical treatment with fluoride gel was measured by a standard method according to ISO 19448:2018 using an Orion 9609BNWP ion-selective electrode (Thermo Fisher Scientific, 22 Alpha Road, Chelmsford, MA 01824, USA).

Prior to the fluoride release measurements and during the measurements (after every 18 readings), the ion-selective electrode was calibrated using commercial standards with a concentration range of 10⁻⁵–10⁻² mol/L F^−^. During the fluoride concentration measurements, the solution was placed on a digital magnetic stirrer RH digital (IKA-Werke GmbH & Co. KG, Staufen, Germany). A sensor was placed in the solution on the magnetic stirrer, which rotated at 500 rpm. An ion-selective electrode connected to a reader Expandable IonAnalyzer EA 940 (Orion Research, 500 Cummings Center Beverly, MA 01915, USA) was immersed in the solution. For each specimen, three readings were taken, from which the mean value of fluoride ion concentration was calculated. After each measurement, the electrode was maintained by removing the sensor, washing it in distilled water, and drying it with paper towels. In the same way, the ion-selective electrode was washed with distilled water and dried.

Because Shapiro–Wilk’s test indicated no significant departures from normality, statistical analysis was performed using parametric tests. The variables “fluoride concentration” and “specimen mass” were compared among materials using one-way ANOVA with Tukey post hoc adjustment for multiple comparisons. The comparisons among time points were performed using repeated-measures ANOVA with Bonferroni post hoc adjustment. The overall level of significance was 0.05. Statistical analysis was performed using the software SPSS, version 25.0 (IBM, 1 New Orchard Rd, Armonk, NY 10504, USA).

## 3. Results

### 3.1. Re-Release of Fluoride Ions

**Figure 2:**
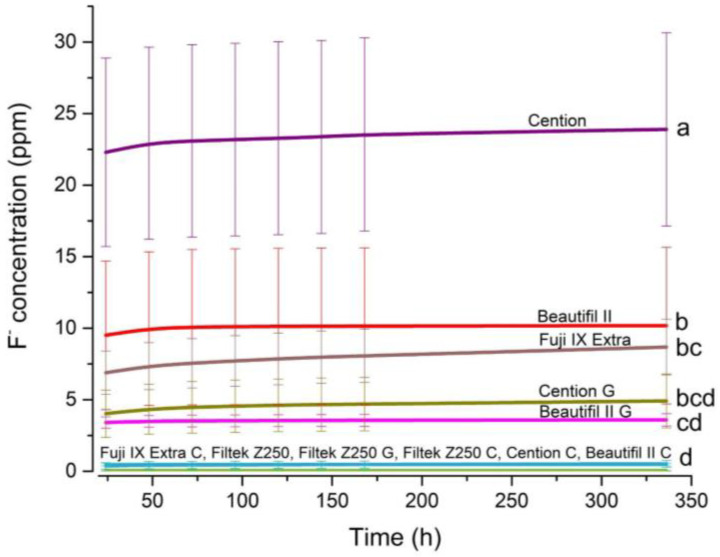
Cumulative concentrations of released fluoride ions measured over 14 days. For statistical comparisons among materials, same letters indicate groups of values that are not significantly different from each other at an overall significance level of 0.05. Error bars denote ± 1 SD. G: specimens coated with G-aenial Bond, C: specimens coated with Clearfil Bond.

The cumulative released fluoride concentrations as a function of time during the 14-day observation period is presented in Figure 2. For the uncoated specimens, the significantly highest released fluoride concentration was achieved by the alkasite composite (Cention) (≈23 ppm), whereas the lowest released fluoride concentrations were observed for the conventional glass-ionomer cement (Fuji IX Extra) (≈7 ppm). The uncoated giomer material (Beautifil II) released significantly less fluoride ions than the uncoated alkasite composite (Cention), whereas fluoride release from uncoated specimens of the giomer (Beautifil Il) and conventional GIC (Fuji IX Extra) was statistically similar. The significantly lowest fluoride re-release among all uncoated materials was observed for the conventional composite material (Filtek Z250).

The comparison of uncoated and coated specimens shows that all coating materials (two adhesive systems and one GIC coating) significantly diminished fluoride re-release. The reduction in fluoride re-release was more pronounced for the specimens coated with the fluoride-releasing universal adhesive (Clearfil Bond) than for the universal one-bottle adhesive (G-aenial Bond, GC Europe, Leuven, Belgium), but the difference was not statistically significant. No effect of adhesive coating on fluoride re-release was observed for the conventional composite material (Filtek Z250), as this material released statistically similar low fluoride concentrations in both uncoated and adhesive-coated specimens. Additionally, all coated specimens, regardless of material and coating type, released statistically similar fluoride concentrations.

**Figure 3:**
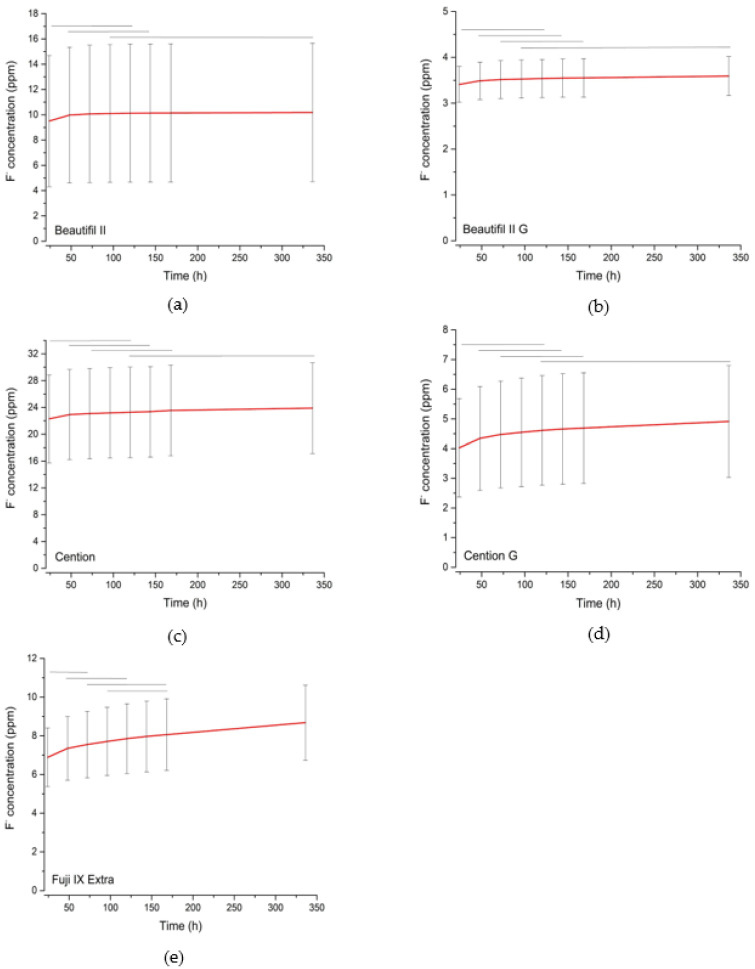
Individual curves of fluoride release with y-axes adjusted to better accommodate the full range of values for each material. Horizontal lines above the curves indicate groups of values that are not significantly different at a 0.05 significance level for statistical comparisons among time points. Error bars denote ± 1 SD. G: specimens coated with G-aenial Bond, C: specimens coated with Clearfil Bond.

Figure 3 shows a magnified view of the individual curves for the five experimental groups with the highest fluoride re-release to illustrate the dynamics of ion release over time and to show the results of the statistical comparisons. All five experimental groups shown reached a plateau within the 14-day observation period, except for the uncoated specimens of the conventional GIC (Fuji IX Extra). For this experimental group, ion release continued beyond the observation period, whereas for all other materials, ion release can be considered to have ceased. The duration of fluoride re-release was estimated from the time required to reach a plateau (Table 2). These values represent the time after which no statistically significant fluoride release was measured.

### 3.2. The Effect of Adhesive/GIC Coating on Fluoride Re-Release 

To quantitatively represent the reduction in fluoride re-release due to specimen coating, a “reduction factor” was calculated by dividing the cumulative fluoride concentrations of uncoated specimens at the end of the 14-day observation period by the corresponding cumulative concentrations of the coated specimens (Table 3). For the alkasite composite (Cention), the presence of a dental adhesive system (Clearfil Bond) reduced fluoride re-release by as much as 8154-fold. For the other restorative materials, the reduction factors ranged from 0.07 to 2431.

### 3.3. Changes in the Mass after the Fluoride Re-Release

**Figure 4:**
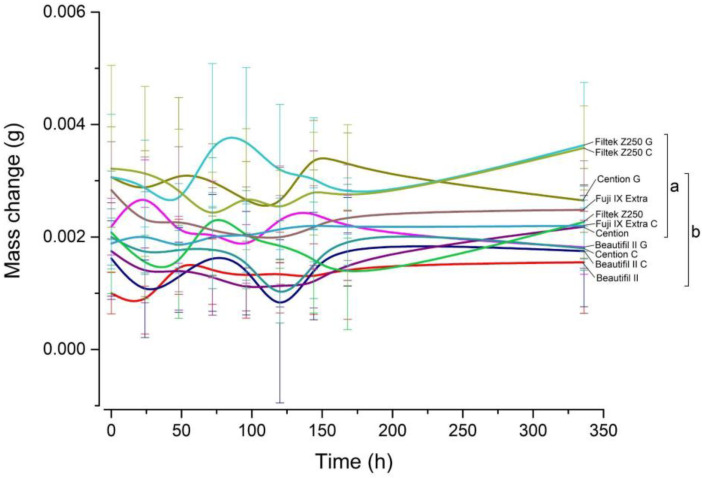
Curves for specimen mass change over time. For statistical comparisons among materials, same letters indicate groups of values that are not significantly different from each other at an overall significance level of 0.05. Error bars denote ± 1 SD.

The changes in specimen mass as a function of time are shown in Figure 4. The comparisons among time points within each material showed no statistically significant mass changes over the observation period. 

## 4. Discussion

Considering that ion-releasing dental materials release fluoride ions only for a limited time [17], it is of interest whether the “depleted” materials can be recharged with fluoride ions. In this way, the effects of recharging could become more useful than the process of initial fluoride release. It has also been suggested that for clinical purposes, it is more important to ensure lower, but sustained, fluoride release over time than to achieve high initial bursts limited to a short period after restoration placement [23].

In the study by Gui et al. [4] the possibility of the initial release and recharge of the material with fluoride ions was investigated. According to their results, glass-ionomer materials showed higher recharge compared to giomer or composite materials [4]. Furthermore, in the study by Naoum et al. [9] the recharge potential was measured using 18-month-old specimens, and it was found that a giomer (Beautifil II) had a comparable long-term fluoride release pattern after recharge treatment as a GIC material (Fuji IX Extra). The giomer showed a higher recharge potential compared to other fluoride-containing composite materials [16]. Naoum et al. [9] conducted another study in which they confirmed the mentioned results.

The results of the aforementioned studies differ from our findings, as we found no statistically significant difference in recharge between a giomer and a glass-ionomer. The differences could be due to the different types of glass-ionomer materials investigated by Gui et al. [4] namely Fuji II LC and Fuji VII. The differences may also be due to variations in topical treatment and fluoride concentrations. Products with higher fluoride concentrations allow greater ion adsorption on the surface of the material [17]. The fluoride gel investigated in our study has a fluoride concentration of 12,300 ppm, whereas in the above-mentioned study [16], the materials were recharged with a NaF solution with a concentration of 5000 ppm.

The differences can also be explained by the age of the material specimens. The specimens in our study were aged for 25 months, whereas in the aforementioned studies the aging periods were 28 days [4], 49 days [9], or 18 months [16]. This assumption is supported by a study [24] showing that the age of glass-ionomer materials can negatively affect the amount of ions that these materials can reabsorb. Although GIC (Fuji IX Extra) has been shown to initially release more fluoride ions than giomer (Beautifil II) in a previous study [9], our results for uncoated specimens showed the lowest cumulative levels of released ions for the GIC compared to the giomer or the alkasite composite. Differences from the aforementioned studies may also lie in the timing of topical treatment. In the study by Bansal and Bansal [20], the specimens were 15 days old when the fluoride treatment was applied. Therefore, we believe that performing topical fluoride treatment while the initial release process has still not been completed may lead to the summation of fluoride ions from the material and ions adsorbed during topical treatment, which then leads to an overestimated fluoride release.

Similar results to ours were reported by Rai et al. [19] who considered alkasite composite (Cention) as a more effective ion-releasing material with the highest initial fluoride release and recharge compared to a giomer (Beautifil II) and a GIC (Fuji IX Extra) [19]. The effective fluoride release and recharge of Cention could be attributed to its three different types of fluorine-enriched fillers: calcium barium aluminum fluorosilicate glass, calcium fluorosilicate, and ytterbium trifluoride [19].

In the study by Mousavinasab and Meyers, a conventional GIC (Fuji IX Extra) released less fluoride ions than a conventional GIC (Fuji VII), which the authors explained by a lower amount of monovalent ions in Fuji IX Extra affecting the polymer chain structure and leading to a lower fluoride release [13].

Our results show that the giomer re-released fewer fluoride ions than the alkasite composite. Accordingly, the first null hypothesis was rejected. The result could be explained by the presence of surface pre-reacted glass-ionomer particles in the giomer; therefore, water sorption does not contribute significantly to fluoride ion release, as shown in a previous study [13]. In the study by Marović et al. [25] alkasite composite (Cention) showed higher solubility than GIC (Fuji IX Extra) and giomer (Beautifil II), whereas giomer showed the lowest water sorption probably due to the high content of filler particles [25]. Considering these results and the fact that our specimens were already aged for 25 months prior to being used in the present study, aging can be hypothesized to have altered the material surface and opened porosities where fluoride ions can be deposited and subsequently released. These considerations may explain why Cention, as the most soluble of all the materials studied [25], re-released the largest amount of ions.

According to Ruengrungsom et al. [2] some glass-ionomer materials showed lower initial fluoride ion release than the previous version of the alkasite composite (Cention N) [2]. The initial higher diffusion of ions could be due to a lower crosslinking density of the resin matrix caused by a low degree of conversion of the dual-curing alkasite [26,27]. Panpisut et al. [28] also investigated the degree of conversion in alkasite composite material (Cention N), resin-modified glass-ionomer material, and conventional composite and showed that Cention achieved lower conversion values than resin-modified GIC [28]. The result of our study—that the alkasite composite showed the highest fluoride release after topical treatment—agrees with the study of Ruengrungsom et al. [2] considering that materials that initially release more ions can also absorb more ions after topical treatment [17].

According to our results, the presence of a dental adhesive system or a GIC coating reduced the recharge capacity even 8154 times compared to the uncoated specimens. This result also led us to reject the first null hypothesis. The adhesive system or GIC coating may harm the recharge process by acting as a barrier to the uptake of fluoride ions. Similar considerations were also expressed in the study by Wang et al. [29] in which they investigated the effects of the coating on initial fluoride release from resin-modified glass-ionomer cements [29]. Another study by Par et al. [30] came to similar conclusions by demonstrating that fluoride release from coated specimens of the alkasite composite (Cention) was reduced 300-fold compared to the uncoated specimens [30]. In contrast to these results, Tay et al. [31] suggested that adhesives may act as permeable membranes that allow remineralization of tooth structure and arrest the caries process. Unlike the inner surface of the restoration, which is adjacent to the cavity wall, the outer restoration surface is exposed to saliva and repeated changes in acidity due to the dynamic oral environment, leading to increased porosity of the material surface over time. These pores could also serve as a pathway for fluoride ion uptake and even surface adsorption due to the enhanced surface roughness [10]. It should be noted that all the above studies have investigated the effects of adhesive systems on initial fluoride release. 

An unexpected finding is that the adhesive system with fluoride release capability (Clearfil Bond) had negative effects on fluoride re-release compared with the universal one-bottle adhesive (G-aenial Bond). This result is following the study by Kelić et al. [22] which also reported lower fluoride release from specimens coated with Clearfil Bond compared to G-aenial Bond. The apparently contradictory results may be explained by the different hydrophilicities of the two adhesive systems which affect their water sorption and may increase permeability over time due to the formation of erosions and cracks [32,33,34]. Whereas G-aenial Bond contains 1–5% phosphoric acid ester monomers, Clearfil Bond contains monomers of hydrophilic amides [35,36]. Ester bonds in resin monomers are prone to hydrolysis [37,38], possibly leading to more surface defects of the G-aenial bond adhesive layer, which in turn improved fluoride uptake and re-release. Additionally, polar groups such as hydroxyl, carboxyl, and phosphate present in G-aenial Bond can form hydrogen bonds with water [36], which may enhance the surface adsorption of fluoride ions. 

Adhesive systems not only reduced the concentration of adsorbed fluoride ions, but the composition of the resin systems could also have played a role in this reduction. The monomers Bis-EMA, Bis-GMA, TEGDMA, and UDMA in the composite material (Filtek Z250) are hydrophobic and have a negative affinity for water sorption. The alkasite material (Cention) contains mainly hydrophobic monomers, but also a hydrophilic monomer PEG-400 DMA, which could explain the significantly higher fluoride re-release for the alkasite composite compared to other materials investigated [39]. 

The rate of re-release was investigated in this study under neutral pH conditions. According to Levallois et al. [40] fluoride release rate is greater under acidic conditions than under neutral conditions. Further, precipitation of fluoride compounds on surface may be highly pH-dependent [41]. To avoid the effect of acidic environment on fluoride precipitation and release, the pH of our study was neutral. The current literature offers a limited number of studies addressing the recharge process of ion-releasing materials. 

Limitations of this study include several topics. The minimum detection level of the ion-sensitive electrode used in this study is 0.02 ppm, according to the manufacturers [42]; therefore, values or differences in that range may not be reliable. The baseline fluoride content of the specimens used in this study was determined in a previous study [22]. The study showed that at the end of the 168-day-trial, released fluoride content was not detectable anymore. Therefore, the baseline released fluoride content for this current study was taken to be as low as undetectable. The study aimed to investigate restorative materials and adhesive treatments effects on fluoride release; therefore, the comparison to enamel or hydroxyapatite discs was not performed. The clinical relevance of applying fluoride to restorative materials and adhesive systems was not observed in this study. Additionally, artificial saliva might contribute to clinical relevance of the results, but in this study, deionized water was used to avoid a slightly reduced ion release affected by artificial saliva [15]. To our knowledge, no research on the recharging of restorative materials has been conducted on in vivo models; therefore, we believe that research on in vivo models should have been conducted before the clinical implementation of the recharging protocol for ion-releasing materials.

## 5. Conclusions

From this in vitro study it can be concluded that:

1. Alkasite composite (Cention), Giomer (Beautiful II) and conventional glass-ionomer cement (Fuji IX Extra) can be recharged with fluoride ions by topically applied NaF gel. Conventional composite (Filtek Z250) showed no recharge ability;
2. The alkasite composite had a better recharge potential than giomer and conventional glass-ionomer cement;
3. Application of the dental adhesive systems and a GIC coating harmed fluoride recharge and re-release.

Further research is needed to confirm these in vitro results and to determine the most beneficial conditions fluoride recharge in a clinical practice.

## Figures and Tables

**Table 1:** The composition of the tested materials provided by the manufacturers.

Material Class	Commercial Name	Composition	Color/LOT No.	Manufacturer	Curing Mechanism
(experimental) Alkasite composite	Cention	Powder: inert barium alumino-boro-silicate glass, ytterbium fluoride, calcium fluoro-alumino-silicate glass, calcium-barium-alumino-fluorosilicate glass Liquid: UDMA, DCP, aromatic-aliphatic-UDMA, PEG-400 DMA; Initiator system: hydroperoxide, Ivocerin and acyl phosphine oxide	A2/ XL7102	Ivoclar Vivadent, Schaan, Lichtenstein	Dual-cure
Giomer	Beautifil II	Fillers: s-PRG (aluminofluoro-borosilicate glass); Resin: bis-GMA, TEGDMA Nano fillers 83.3 wt%	A2/051829	Shofu Dental GmbH, Ratingen, Germany	Light-cure
Glass-ionomer cement	Fuji IX Extra	Powder: fluoro-alumino-silicate glass Liquid: 5–10% polybasic carboxylic acid (copolymer of acrylic and maleic acid), tartaric acid, water	A3/1801171	GC Europe, Leuven, Belgium	Self-cure
Conventional composite	Filtek Z250	Filler: zirconia and silica particles Resin: bis-GMA, TEGDMA, UDMA; 78.5 wt% 60% vol.	A2/N984652	3M Deutschland GmbH, Neuss, Germany	Light-cure
Universal adhesive	G-aenial Bond	acetone: 25–50%; dimethacrylate: 10–20%; phosphoric acid ester monomer: 5–10%; dimethacrylate component: 1–5%; photoinitiator: 1–5%; polymerization inhibitor: BHT < 1%	1811281	GC Europe, Leuven, Belgium	Light-cure
Universal fluoride- releasing adhesive	Clearfil Universal Bond Quick	ethanol 10–25% monomer: bis-GMA 10–25%, hydroxyethylmethacrylate 2.5–10%, methacryloyloxydecyl dihydrogen phosphate, hydrophilic amide monomers; colloidal silica, silane coupling agent; sodium fluoride; photoinitiator: camphorquinone; water	3L0108	Kuraray Europe, Hattersheim am Main, Germany	Light-cure
Glass-ionomer coat	GC Fuji Coat LC	Monomer: MMA 25–50%; Photoinitiator: 1–5%; polymerization inhibitor: BHT < 1%	1804021	GC Europe, Leuven, Belgium	Light-cure

UDMA: urethane dimethacrylate; DCP: Tricyclodecandimethanol dimethacrylate; PEG-400 DMA: Polyethylene glycol 400 dimethacrylate; s-PRG: surface pre-reacted glass-ionomer; bis- GMA: Bisphenol A diglycidyl ether dimethacrylate; TEGDMA: triethylene glycol dimethacrylate; BHT: butylated hydroxytoluene; MMA: methyl methacrylate.

**Table 2:** Time to reach a plateau in fluoride concentration curves, defined as the time point after which successive values remained statistically similar.

Material	Time (Days)
Beautifil II	4
Beautifil II G-aenial	4
Cention	5
Cention G-aenial	5
Fuji IX Extra	>14

**Table 3:** Reduction factors calculated by dividing the cumulative fluoride release of the uncoated specimens by the corresponding values of the coated specimens.

Uncoated/Coated Specimens	Reduction Factor
B/BG	2.83
B/BC	2431.30
C/CG	4.86
C/CC	8154.64
Z/ZG	0.07
Z/ZC	16.40
F/FC	16.82

B—Beautifil II; BG—Beautifil II coated with G-aenial Bond; BC—Beautifil II coated with Clearfil Bond; C—Cention; CG—Cention coated with G-aenial Bond; CC—Cention coated with Clearfil Bond; Z—Filtek Z250; ZG—Filtek Z250 coated with G-aenial Bond; ZC—Filtek Z250 coated with Clearfil Bond; F—Fuji IX Extra; FC—Fuji IX Extra coated with Fuji IX Extra Coat GP.

## Data Availability

Data supporting reported results can be reached from the corresponding author, upon a reasonable request.
